# *Zingiber officinale* Roscoe Rhizome Extract Exerts Senomorphic and Anti-Inflammatory Activities on Human Endothelial Cells

**DOI:** 10.3390/biology12030438

**Published:** 2023-03-12

**Authors:** Giulia Matacchione, Vittoria Borgonetti, Deborah Ramini, Andrea Silvestrini, Marta Ojetti, Nicoletta Galeotti, Fabiola Olivieri

**Affiliations:** 1 Department of Clinical and Molecular Sciences, Università Politecnica Delle Marche, Via Tronto 10/A, 60126 Ancona, Italy; 2 Department of Neuroscience, Psychology, Drug Research and Child Health (NEUROFARBA), Section of Pharmacology and Toxicology, University of Florence, Viale G. Pieraccini 6, 50139 Florence, Italy; 3 Clinic of Laboratory and Precision Medicine, IRCCS INRCA, via Birrarelli 8, 60121 Ancona, Italy

**Keywords:** senescence, inflammaging, natural compounds, senomorphics, neuroinflammation, Zingiber officinalis roscoe

## Abstract

**Simple Summary:**

Aging is related to a low-grade and sterile inflammation called inflammaging, recognized as the main risk factor for age-related disease (such as diabetes, dementia, and cancer) development. At present, several natural compounds have gained attention to be tested in the framework of anti-aging therapies. Here, we investigated the anti-senescence and anti-inflammatory properties of an Asian-native Ginger extract, commonly consumed as a food spice and herbal medicine, on human endothelial cells and murine microglial cells. The anti-senescence and anti-neuroinflammatory effects that we observed on such cellular models suggest the potential relevance of Ginger extract in delaying/postponing the most common age-related diseases development and progression.

**Abstract:**

Aging is related to a low-grade and sterile inflammation called inflammaging, recognized as the main risk factor for age-related disease (ARD) development. Inflammaging is fostered by the repeated activation of immune cells, as well as by the accumulation of senescent cells. Recently, a number of natural compounds have gained attention to be tested as anti-aging therapies, based on their anti-inflammatory activity and/or ability to reduce the pro-inflammatory secretome of senescent cells (senomorphyc activity). Here, we investigated the anti-inflammatory and senomorphic properties of an Asian-native *Zingiber officinale* Roscoe extract (ZOE), commonly consumed as a food spice and herbal medicine. We employed two models of primary endothelial cells (HUVECs), such as the replicative-senescence and LPS-induced response, to investigate the anti-inflammatory/senomorphic effect of ZOE, and one cellular model of neuroinflammation, i.e., immortalized murine microglial cells (BV2). First, we found that the ZOE treatment induced the inhibition of NF-kB activation in BV2 cells. Among the constituents of ZOE, we showed that the terpenoid-enriched fraction (ZTE) was the component able to counteract the phosphorylation of NF-kB(p65), while 6-gingerol (GIN) and 6-shogaol (SHO) did not produce any significant effect. Further, we observed that the treatment with 10 µg/mL of ZOE exerted anti-inflammatory activity on LPS-stimulated young (y)HUVEC and senomorphyc activity on replicative senescent (s)HUVEC, significantly reducing the expression levels of IL-1β, TNF -α, IL-8, MCP-1, and ICAM-1. Moreover, the ZTE treatment was able to significantly reduce the IL-8 levels secreted in the medium of both LPS-stimulated yHUVEC and sHUVEC. Overall, our data suggest a potential protective role of ZOE on neuroinflammation and endothelial inflammation/activation, thus suggesting its potential relevance in delaying/postponing ARD development and progression, characterized by endothelial dysfunction.

## 1. Introduction

Chronic, low-grade, and sterile pro-inflammatory status is a pervasive phenomenon of aging. It has been defined as “inflammaging”, and it was identified as an important contributor to tissue dysfunction and a significant risk factor for the development of the most common age-related diseases (ARDs), such as diabetes, neurodegenerative diseases, and atherosclerosis [1,2]. Overall, inflammaging level is currently recognized as the main risk factor for morbidity and mortality in the elderly [3,4]. Inflammaging is fostered by the repeated activation of the immune cells, as well as by the accumulation of senescent cells during aging. Senescent cells are characterized by cell cycle arrest in association with alterations in the metabolic activities and morphology. The most relevant senescent-associated feature in the framework of inflammaging is the development of a pro-inflammatory secretory phenotype, named senescence-associated secretory phenotype (SASP) [5,6].

Eliminating senescent cells has been shown to improve age phenotypes in mouse models [7], and there is some initial evidence that it may contribute to postponing ARDs, thus extending the health span [2]. Senomorphic treatment provides an alternative pharmacological approach to targeting senescent cells, as it can suppress the detrimental effects of SASP, without affecting cell viability [8]. At the molecular level, senomorphics act by targeting the most relevant transcription factors, i.e., NF-kB, for inflammatory mediators, which are released as SASP factors (such as cytokines, chemokines, and metalloproteases).

In the research of senolytic/senomorphyc compounds, the attention of the scientific community was attracted by natural compounds, used in ancient medicine, known for their beneficial effects and high tolerability. The Asian-native *Zingiber officinale* Roscoe, commonly known as ginger, has long been widely consumed as a food spice and herbal medicine to treat various symptoms, including vomiting, nausea, and pain [9,10].

Biological compounds are contained in the dried rhizome, which is particularly abundant in essential oil and oleoresin. The non-volatile components, which represent the phenolic compounds, include gingerols, shogaols, paradols, and zingerone [11]. 

Among terpenes, zingiberene is the major component found in ginger root and has been known for its anti-bacterial activities, anti-carcinogenic properties, and help in preventing the high blood sugar levels that are often associated with its anti-oxidative and anti-inflammatory activities [12]. Moreover, ginger extracts were studied to treat neuropathic pain symptoms in animal models by inhibiting neuroinflammation [13]. 

In addition, recent evidence has highlighted the ability of ginger extracts to restrain inflammation, also by targeting senescent cells [14]. Among the components of ginger extract, gingerenone A and 6-shogaol showed promising senolytic properties, characterized by the selective elimination of senescent cells [14].

The central role played by the endothelium and innate immune cells in activating and sustaining acute and chronic inflammation justifies the efforts to identify new strategies to counteract endothelial cell degeneration and immune cell activation. In this framework, we aimed to analyze, in vitro, the anti-inflammatory effects of ginger extracts and some of its components on both microglia and endothelial cells. Considering the crucial role of endothelial dysfunction and neuroinflammation during aging and ARDs, we employed two models of primary endothelial cells (HUVECs), replicative-senescence and LPS-induced response, to investigate the possible anti-inflammatory/senomorphic effect of ginger extracts, in addition to one cellular model of neuroinflammation, i.e., immortalized murine microglial cells (BV2). 

We tested a standardized *Z. officinale* rhizomes extract (ZOE), obtained by CO_2_ supercritical extraction on both cellular models of microglia and endothelial cells. In particular, 6-gingerol (GIN), 6-shogaol (SHO), and terpenoid-enriched fraction (ZTE), were tested on the microglia. As the volatile component of ZOE, ZTE, which contains Zingiberene, was the most effective in restraining inflammation in the immune cells, we also tested its effect on the endothelial cells. 

## 2. Materials and Methods

### 2.1. Chemicals

The ZOE, obtained by supercritical CO_2_ extraction, and standardized to contain 24.73% total gingerols and 3.03% total shogaols, was kindly provided by INDENA S.p.A. (Milan, Italy), batch number 46349. The 6-gingerol (GIN) was purchased from Sigma-Aldrich (Milan, Italy) and the 6-shogaol (SHO) was purchased from Extrasynthese (Genay, France). The extraction of the terpenoid-enriched fraction (ZTE) was performed as previously described (Ferguson, 1956). The quantification of GIN and SHO in the ZOE was obtained by HPLC-DAD, while the volatile compounds were analyzed through gas chromatography coupled with a flame ionization detector (GC-FID) and with a mass spectrometer, as previously reported [13]. 

### 2.2. BV2 Cells

Immortalized murine microglial cells (BV2; C57BL/6 Tema Ricerca, Genova, Italy) were cultured in 75 cm^2^ flasks (Sarstedt, Verona, Italy) in a medium containing RPMI with 10% heat-inactivated fetal bovine serum (56 °C, 30 min) (FBS, Gibco^®^, Milan, Italy), 1% glutamine, and 1% penicillin-streptomycin solution (Merck, Milan, Italy). The cells were cultured at 37 °C and 5% CO_2_ with a daily change of the culture medium.

### 2.3. Neuroinflammatory Model

For the neuroinflammatory model, bacterial lipopolysaccharide from Gram- (LPS, Salmonella enteritidis, Merck, Darmstadt, Germany) was solubilized in RPMI to obtain a 500 µg/mL stock, which was then diluted in the medium to obtain a final concentration of 250 ng/mL. BV-2 cells (3  ×  10^5^ cells/well) were seeded into 6-well plates and cultured for 24 h. We previously performed a dose-response curve, obtained using a CCK-8 assay, to investigate the maximum non-cytotoxic concentration of ZOE on the BV2 cells [13]. Briefly, the cells were treated with increasing concentrations of ZOE (i.e., 0.01, 1, 5, 10, 50, and 100 μg/mL) for 24 h. The maximum non-cytotoxic concentration was found to be 10 μg/mL. The concentrations of GIN, SHO, and ZTE were calculated by considering the percentage of each compound within the extract. Thus, the cells were pre-treated for 4 h with ZOE (10 μg/mL), GIN (1 μg/mL), SHO (0.17 µg/mL), and ZOE terpenoid-enriched fraction (ZTE, 3 µg/mL), and then stimulated with LPS (250 ng/mL) for 24 h [15].

### 2.4. HUVEC Cells

Human umbilical vein endothelial cells (HUVEC) are a pool of endothelial primary cells derived from the human umbilical vein (Clonetics, Lonza, Switzerland). The HUVECs were cultured in an endothelial growth medium (EGM-2, Lonza, Switzerland), composed of endothelial basal medium (EBM-2, Lonza, Switzerland) and the SingleQuot Bullet Kit (Lonza, Switzerland). The HUVECs were seeded at a density of 5000/cm^2^ in T75 flasks (Corning Costar, Sigma Aldrich, St. Louis, MO, USA) and cultured at 37 °C and 5% CO_2_ with a daily change of the culture medium.

### 2.5. Characterization of Young and Senescent HUVEC Cells 

The senescent phenotype was acquired by replicative exhaustion and measured as through cumulative population doublings (cPD). The cPD is described as the sum of the PD, which is measured by the formula: (log_10_(F)–log_10_(I))/log_10_(2), where F is the number of cells at the end of the passage, and I is the number of seeded cells. 

HUVECs were considered young or senescent based on three different markers of senescence: the cPD, the senescence-associated (SA)-β -Galactosidase (β-Gal) activity, and the expression of p16^ink4a^. The SA-β-Gal activity was detected using a Senescence Detection Kit (BioVision Inc., Milpitas, CA, USA), following the manufacturer’s instructions.

### 2.6. Cell Viability Assay

We tested the cell viability using the MTT (3-(4,5-dimethylthiazol-2-yl)-2,5-diphenyltetrazolium bromide) assay. Young (y) and senescent (s) HUVECs were grown in 24-well plates at a density of 5 × 10^4^ cells/cm^2^ and then treated for 24 h with different doses of ZOE.

Briefly, the MTT (1 mg/mL) solution was added, and the cells were incubated for 4 h; the obtained product, a formazan salt, was solubilized in dimethyl sulfoxide (DMSO) and measured by a microplate reader (MPT Reader, Invitrogen, Milano, Italy) at the optical density of 540 nm.

The cell viability was calculated according to the equation (T/C) 100%, where T and C represent the mean optical density of the treated group and the control group, respectively.

### 2.7. Zingiber Officinale Roscoe Extract Treatments

The yHUVEC were pretreated with ZOE (10 µg/mL) or ZTE (3 µg/mL) for 4 h, followed by 24 h treatment with LPS (from *E. coli* O55:B5, Sigma Aldrich, St. Louis, MO, USA) to induce inflammation, together with ZOE (10 µg/mL) or ZTE (3 µg/mL). The sHUVEC were treated with ZOE (10 µg/mL) or ZTE (3 µg/mL) for 24 h. The HUVECs were grown in EGM-2 medium as a control (yHUVEC or sHUVEC). Following these treatments, the cell pellets were prepared and used for the subsequent analysis.

### 2.8. RNA Isolation, mRNA and Mature miRNAs Expression by RT-qPCR

The Total RNA Norgen Biotek Kit (Thorold, ON, Canada) was used to isolate the RNA according to the manufacturer’s instructions, and these were stored at −80 °C until use. The mRNA and miRNA expression were determined through RT-qPCR analysis, as previously described [16]. Briefly, the PrimeScript™ RT reagent Kit with gDNA Eraser and the TB Green™ Premix Ex Taq™ (TAKARA, Shiga, Japan) were used to respectively reverse-transcribe and amplify the mRNA, whereas the TaqMan miRNA assay (ThermoFisher Scientific, Waltham, Massachusetts, United States) was employed for the miRNA quantification. The RT-qPCR analysis was standardized with GAPDH and β-actin for the mRNA expression and with RNU48 for the miRNA expression.

### 2.9. Western Blot Analysis

The HUVECs were lysed with the RIPA solution (0.1% SDS, 150 mM NaCl, 1.0% Triton X-100, 10 mM Tris, 5 mM EDTA pH 8.0), to which we added a protease and phosphatase inhibitor cocktail (Roche Applied Science, Indianapolis, IN, USA). In each sample, the protein concentration was evaluated using a Bradford assay. Proteins (25 µg) were separated by 4–15% precast polyacrylamide gel (Bio-Rad, Hercules, CA, USA), and then transferred to a 0.2 mm nitrocellulose membrane (Bio-Rad, Hercules, CA, USA). A blocking buffer (Bio-Rad, Hercules, CA, USA) was used to block the membrane, which was then incubated overnight with primary antibodies. Mouse anti-phospho-p38 (Cell Signaling), rabbit anti-phospho-NF-kB (Cell Signaling), mouse anti-ICAM-1 (Cell Signaling), mouse anti-β-actin, mouse anti-α-tubulin, and rabbit-anti-GAPDH (Cell Signaling) were used as the primary antibodies. Anti-mouse or anti-rabbit horseradish peroxidase-conjugated antibodies were used as the secondary antibodies (The Jackson laboratory, Bar Harbor, ME, USA). A Uvitec Imager (UVItec, Cambridge, UK) was used to distinguish the protein bands, using a chemiluminescence substrate (Bio-Rad), that were then quantified using ImageJ software. Each measure was normalized versus β-actin, α-tubulin, or GAPDH.

### 2.10. ELISA Assay

A conditioned medium was collected from the BV-2 and HUVEC cultures at the end of each incubation, centrifuged at 14.000 RPM for 20 min, and then stored at −80 °C until use. An IL-8, TNF-α, and IL-1β ELISA Kit (Invitrogen, Waltham, MA, USA) was used to measure the concentration of these cytokines released in the medium, according to the manufacturer’s instructions.

### 2.11. Statistical Analysis

The reported data are indicated as the mean of three independent replicates ± SD or frequency (%). A paired sample T-test was used for the analysis of the real-time, densitometric data and the ELISA. Data analysis was performed using IBM SPSS Statistics for Windows, version 25 (IBM Corp, Armonk, NY, USA). Statistical significance was defined as a two-tailed *p*-value < 0.05.

## 3. Results

### 3.1. ZTE Is the Main Responsible for ZOE Anti-Inflammatory Activity in the In Vitro Model of Neuroinflammation

**Figure 1:**
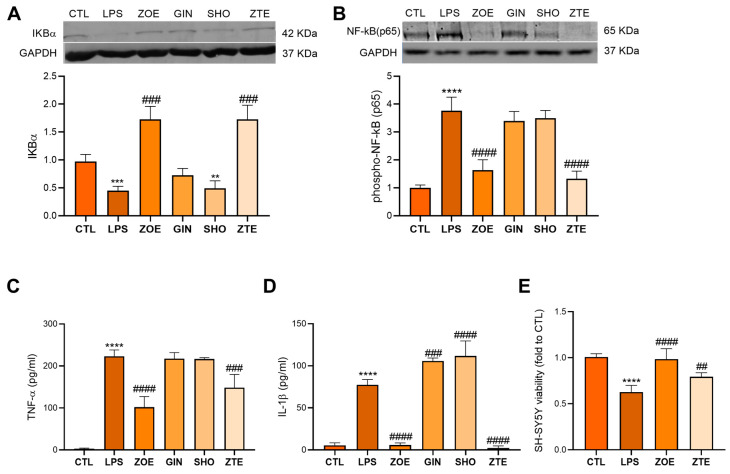
Anti-inflammatory effects of ZOE and ZTE in BV2 cells. Modulation by ZOE (10 µg/mL), GIN (1 µg/mL), SHO (0.17 µg/mL) and ZTE (3 µg/mL) of IKB-α (**A**) and phospho-NFkBp65 (**B**) protein levels in LPS-stimulated BV2 cells. Effect of ZOE (10 µg/mL), GIN (1 µg/mL), SHO (0.17 µg/mL) and ZTE (3 µg/mL) on the release of TNF-α (**C**) and IL-1β (**D**) by LPS-stimulated BV2 cells. Protective effect of ZOE (10 μg/mL), and ZTE (3 μg/mL) on the neurotoxic effect induced by LPS-conditioned BV2 medium (**E**). Asterisks (*) indicate significance versus CTL; (#) indicates significance versus BV2 + LPS; two symbols, *p* < 0.01; three symbols, *p* < 0.001; four symbol, *p* < 0.0001.

The anti-neuroinflammatory activity of ZOE has recently been described [13]. To identify which constituent of ZOE was the most responsible for these effects, we tested the activity of GIN, SHO, and ZTE, at the concentration present in the active dose of ZOE, on the protein activation of NF-kB and cytokines expression. The phosphorylation of the NF-kB-p65 subunit represents one of the most important pathways involved in the inflammatory processes [17]. The kinase IKB-α is a main regulator of the NF-kB signaling pathways, as its degradation allowed the NF-kB-p65 subunit to enter into the nucleus and regulate the target gene transcription [18]. Therefore, we studied its role in the mechanism of action of ZOE. The treatment with ZOE 10 µg/mL up-regulated the levels of IKB-α and reduced the phosphorylation of NF-kB(p65) in the LPS-stimulated BV2 cells. Among the treatments, ZTE was the fraction able to counteract the downregulation of IKB-α and the phosphorylation of NF-kB(p65), while GIN and SHO did not produce any effect (Figure 1A,B). The activation of this transcription factor results in its nuclear translocation and in the increased expression of the genes involved in the inflammatory processes, including the pro-inflammatory cytokines, tumor necrosis factor α (TNF-α) (Figure 1C), and interleukin-1β (IL-1β) (Figure 1D) that were overexpressed in the culture medium of BV2 stimulated with LPS. The pretreatment with ZOE and ZTE strongly prevented these events. In contrast, GIN and SHO showed no significant effect. The inflammatory factors released by the pro-inflammatory microglia are known to induce neurotoxicity; in fact, the LPS-conditioned BV2 medium reduced the viability of SH-SY5Y cells compared to the untreated control. The LPS-conditioned BV2 medium from the cells treated with ZOE and ZTE completely prevented this cytotoxic effect, returning to the control values (Figure 1E). Thus, the anti-neuroinflammatory activity of ZOE appears to be prominently related to its content in ZTE.

### 3.2. Replicative Senescence in HUVECs

**Figure 2:**
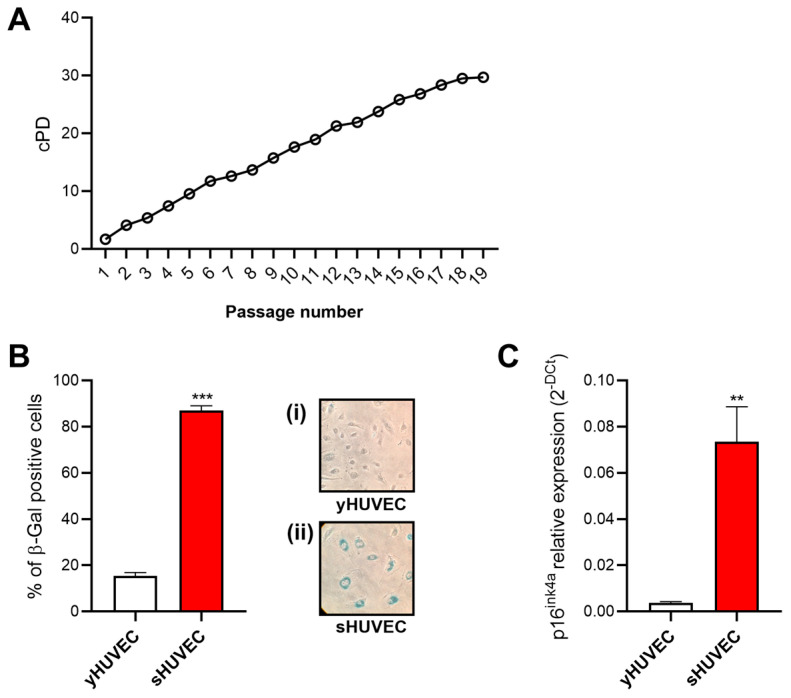
Replicative senescence in HUVECs. Characterization of sHUVEC by a cumulative population doubling curve (**A**), β-Gal activity (**B**) in yHUVEC (i) and sHUVEC (ii) and p16ink4a expression level (**C**). The results are expressed as mean ±SD from three independent biological replicates. **, *p* < 0.01; ***, *p* < 0.001.

To assess the capacity of ZOE and ZTE to reduce inflammation and SASP, we first described yHUVEC and sHUVEC. The yHUVEC were characterized by cPD < 8 and SA-β-Gal positive cells < 15%, whereas the sHUVECs were characterized by cPD > 16 and SA-β-Gal positive cells > 80% (Figure 2A,B). In addition, a significant increase in the p16^ink4a^ mRNA (Figure 2C) levels in the sHUVEC compared to the yHUVEC confirm the acquisition of the senescent status. 

### 3.3. Zingiber Officinale Extract Effect on HUVEC Viability

**Figure 3:**
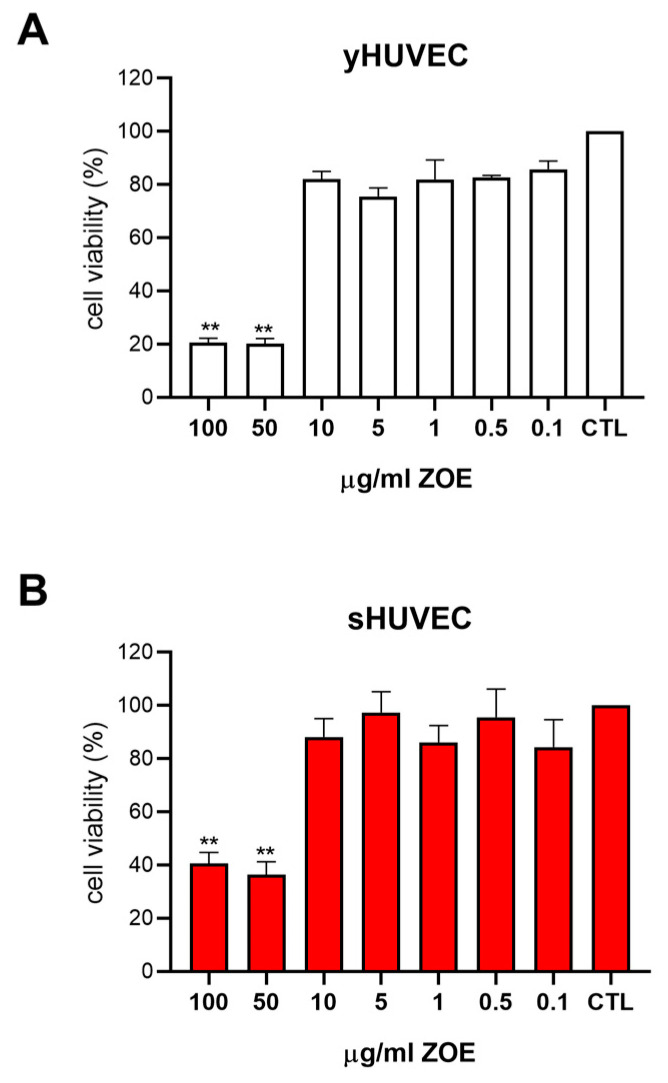
Dose–response curve of HUVEC to *Zingiber officinale* extract (ZOE). yHUVEC (**A**) and sHUVEC (**B**) were treated with different concentrations of ZOE (from 0.1 µg/mL to 100 µg/mL). Histograms indicate the percentage of cell viability normalized to the viability of DMSO-treated cells (CTL) and presented as mean value ±SD from three independent biological replicates. **, *p* < 0.01.

The different ZOE concentrations (100, 50, 10, 5, 1, 0.5, 0.1 µg/mL) were evaluated using the MTT (3-(4,5-dimethylthiazol-2-yl)-2,5-diphenyltetrazolium bromide) assay the yHUVEC and sHUVEC after 24 h of treatment (Figure 3A,B). The experiments were then performed considering the concentration of the extract that gave at least 80% viability of the treated HUVEC (10 µg/mL ZOE). Subsequently, the HUVEC were treated with 3 µg/mL ZTE, which corresponds to 30% of the tested ZOE concentration.

### 3.4. ZOE Anti-Inflammatory Activity in LPS-Stimulated HUVECs

**Figure 4:**
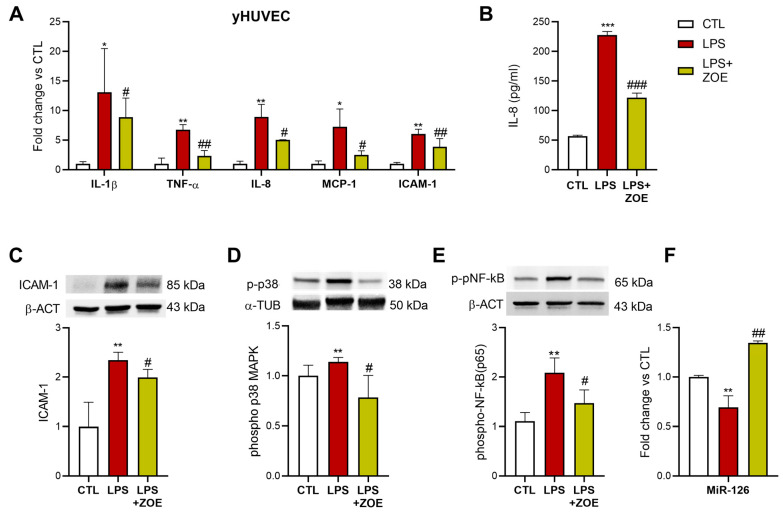
Effect of ZOE on yHUVEC stimulated with LPS. Relative mRNA expression of IL-1β, TNF-α, MCP-1, IL-8, and ICAM-1 (**A**), concentration (pg/mL) of IL-8 in the culture medium (**B**), representative western blot analysis showing ICAM-1 (**C**), p-p38 MAPK (**D**) and p-NF-kB (**E**) expression and expression levels of miR-126 (**F**) in yHUVEC. β-actin and α-tubulin were used as a control. The bands were quantified by ImageJ. All data are reported as fold change vs. untreated yHUVEC. The results are expressed as mean ±SD from three independent biological replicates. Asterisks (*) indicate significance versus CTL; (#) indicates significance versus yHUVEC + LPS; one symbol, *p* < 0.05; two symbols, *p* < 0.01; three symbols, *p* < 0.001.

The potential anti-inflammatory capacity of ZOE was evaluated by assessing the expression of pro-inflammatory markers in the yHUVEC stimulated with 250 ng/mL LPS for 24 h. The yHUVEC were pretreated for 4 h with ZOE and then 24 h with ZOE and LPS. The ZOE significantly decreased the expression levels of the cytokines IL-1β and TNF -α, the chemokines IL-8 and MCP-1, and the adhesion molecule ICAM-1 compared to the LPS-stimulated HUVEC (Figure 4A). Furthermore, the release of IL-8 in the medium (Figure 4B) in the LPS-stimulated HUVEC was significantly reduced by the ZOE treatment. The ZOE treatment was also associated with a significant down-regulation of the ICAM-1 protein level (Figure 4C), a significant reduction in the phospho-p38 MAP kinase (MAPK) activation (Figure 4D), and a significative down-regulation in the NF-kB p65 subunit phosphorylation (Figure 4E), compared to the LPS-stimulated HUVECs. Finally, we analyzed the expression of the *angio-miR*, miR-126, one of the main regulators of angiogenesis and endothelial proliferation [19]; MiR-126 was significantly up-regulated in the LPS-stimulated cells treated with ZOE (Figure 4F).

### 3.5. Senomorphic Effect of ZOE in Senescent HUVECs

**Figure 5:**
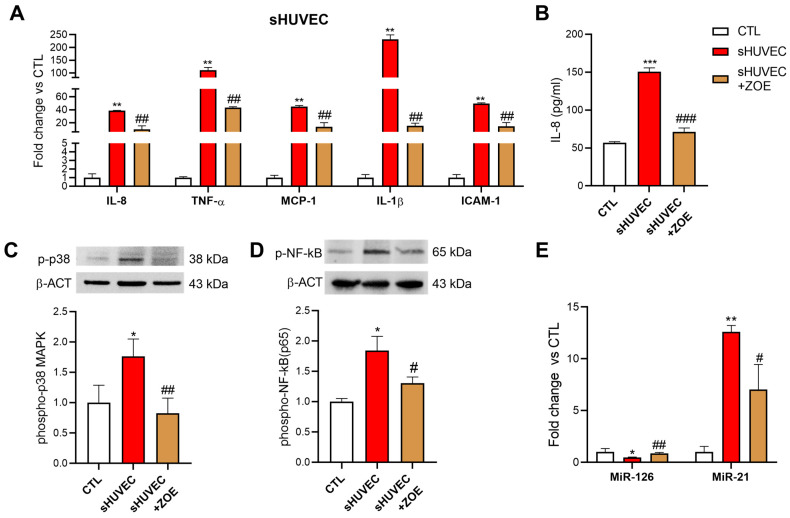
ZOE senomorphic effect on sHUVEC. Relative mRNA expression of IL-1β, TNF-α, MCP-1, IL-8, and ICAM-1 (**A**), concentration (pg/mL) of IL-8 in the culture medium (**B**), representative western blot analysis showing phospho-p38 MAPK (**C**) and phospho-NF-kB(p65) (**D**) expression level in yHUVEC, expression levels of miR-21 and miR-126 (**E**). β-actin and α-tubulin were used as a control. The bands were quantified by ImageJ. All data are reported as fold change vs. yHUVEC (CTL). The results are expressed as mean ±SD from three independent biological replicates. Asterisks (*) indicate significance versus CTL; (#) indicates significance versus untreated sHUVEC; one symbol, *p* < 0.05; two symbols, *p* < 0.01; three symbols, *p* < 0.001.

To evaluate the potential senomorphic activity of ZOE, we analyzed the expression levels of several molecules, considered as SASP components or modulators, such as proinflammatory cytokines and chemokines (IL-1β, TNF-α, IL-8, and MCP-1), adhesion molecules (ICAM-1), microRNAs (*inflamma-miR*-21 and *angio-miR-*126), and transcription factors (NF-kB). The sHUVEC were treated with 10 µg/mL of ZOE for 24 h. The expression of TNF-α, IL-8, MCP-1, IL-1β, and ICAM-1, as well as the expression of miR-21, were significantly increased in the sHUVEC compared to the yHUVEC. Interestingly, the ZOE treatment of sHUVEC was able to significantly decrease the TNF-α, IL-8, MCP-1, IL-1β, and ICAM-1 expression levels (Figure 5A). The ZOE treatment was able to induce an important reduction in the IL-8 chemokine release in the culture medium (Figure 5B). Regarding p38 MAPK and NF-kB activation, in the sHUVEC treated with ZOE, the expression of both phospho-p38 MAPK and phospho-NF-kBp65 were downregulated compared to the non-treated senescent cells (Figure 5D). Moreover, the ZOE treatment was associated with a strong down-regulation of miR-21 and with an up-regulation of the miR-126 expression levels (Figure 5E).

### 3.6. ZTE Biological Activity in yHUVEC and sHUVEC

**Figure 6:**
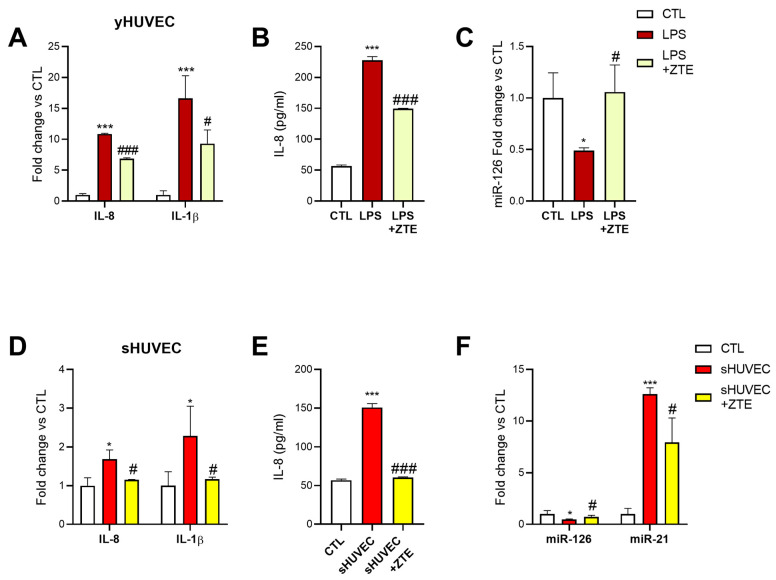
Effect of ZTE on HUVEC. Histograms represent IL-1β and IL-8 mRNA level (**A**), IL-8 release in the culture medium (**B**) and miR-126 expression (**C**) in yHUVECs; IL-1β and IL-8 mRNA level (**D**), IL-8 release in the culture medium (**E**) and miR-126 and miR-21 expression (**F**) in sHUVECs. All data are reported as fold change vs. untreated yHUVEC (CTL). The results are expressed as mean ±SD from three independent biological replicates. Asterisks (*) indicate significance versus CTL; (#) indicates significance versus yHUVEC+LPS or untreated sHUVEC; one symbol, *p* < 0.05; three symbols, *p* < 0.001.

ZTE is a volatile component of ZOE, composed of 28.2% of zingiberene, which is the most abundant volatile compound. We observed that ZTE was the ZOE fraction responsible for its anti-neuroinflammatory activity (Figure 1). To assess whether ZTE is responsible for ZOE’s anti-inflammatory and senomorphic effects, we tested the activity of ZTE in the LPS-stimulated yHUVEC and in the sHUVEC at the concentration of 3 µg/mL (30% of the dose of ZOE) on the pro-inflammatory markers. The treatment with ZTE in the yHUVEC was associated with a significant reduction in the IL-1β and IL-8 mRNA expression (Figure 6A), IL-8 secretion (Figure 6B), and miR-126 level (Figure 6C) compared to the LPS-treated yHUVEC.

Similarly, the sHUVEC treated with ZTE showed a significant down-regulation of IL-1β and IL-8 mRNA expression (Figure 6D), as well as IL-8 secretion (Figure 6E) and the miR-126 and miR-21 levels (Figure 6F) compared to the sHUVEC.

## 4. Discussion 

Aging is described as the progressive loss of physiological integrity associated with functional impairment and a high susceptibility to several pathologies, defined as age-related diseases (ARDs) [1,20]. The accumulation of the senescent cells that acquire SASP is now recognized as one of the main detrimental characteristics of the aging process, fostering inflammation, and thus promoting degeneration and tumorigenesis [14]. SASP factors consist of a plethora of pro-inflammatory cytokines, chemokines, growth factors, and matrix-remodeling enzymes that affect the microenvironment surrounding the senescent cells [21]. The central role played by the vasculature and innate immunity in activating and sustaining acute and chronic inflammation justifies the efforts to identify new strategies to counteract endothelial degeneration and immune cell activation. 

Although the anti-inflammatory activity of *Zingiber officinale* roscoe (ZOE) has been already well recognized in both in vivo and in vitro models [14,22,23,24,25], studies on the senomorphic activity in human endothelial cells, as well as on neuroinflammation, are scarce. 

First, we found an anti-neuroinflammatory activity of ZOE in the BV2 murine microglial cells, which was perpetuated at the molecular level by the inhibition of NF-kB activation. As NF-kB signaling is partly responsible for the onset of neuropathic symptoms, ZOE could be suggested as a nutraceutical compound to control neuropathy.

The primary human endothelial cells obtained from the umbilical vein (HUVEC) were extensively investigated as a human model of endothelial cells to assess the effects of senolytic and senomorphic compounds [26,27,28,29]. We observed that 10 µg/mL of ZOE exerts anti-inflammatory activity on LPS-stimulated yHUVEC by reducing the expression levels of IL-1β, IL-8, MCP-1, TNF-α, and ICAM-1. ICAM-1 reduction was also confirmed at the protein level, suggesting that ZOE could impair leucocyte adhesion to endothelial cells and transmigration through the vasculature. Moreover, we examined the activation of p38 MAP kinase (MAPK) and the p65 subunit of NF-kB.

Both the key MAPK p38 and the nuclear transcription factor NF-kB are involved in regulating inflammation and in the production of inflammatory mediators [17,30]. 

The ZOE treatment of the LPS-stimulated HUVEC was associated with a down-regulation of p38-MAPK activation, through the phosphorylation of threonine 180 and tyrosine 182, as well as the reduction in the NF-kB-p65 subunit phosphorylation (serine 536).

The senomorphic effect of ZOE in the replicative senescent HUVEC was demonstrated by the reduced expression of pro-inflammatory markers, such as IL-8, IL-1β, MCP-1, TNF-α, and ICAM-1. 

Importantly, the ZOE treatment was found to decrease the level of the phosphorylated p65 subunit of the NF-kB in the sHUVEC. NF-kB is one of the master regulators of the inflammatory process and activates the target genes that could contribute to cellular senescence and SASP acquisition [31]. NF-kB aberrant activation was associated with ARD development and progression [32]. In this framework, the senotherapeutic properties of ZOE suggest a potential contribution to reducing the progression rate of the most common human ARDs, such as diabetes and neurodegenerative and cardiovascular diseases. Some evidence on this topic has already been provided. Ginger supplementation of type 2 diabetic patients [33], as well as of patients affected by osteoarthritis [34], was associated with reduced inflammatory cytokines. 

We have also analyzed the expression levels of two miRNAs: miR-21, involved in the modulation of inflammatory pathways; and miR-126, previously associated with vascular development and homeostasis [35,36]. To the best of our knowledge, there is still no evidence regarding the role of ZOE in modulating miRNA expression in HUVECs. Here, we found that the levels of miR-126 were up-regulated by ZOE treatment in both the yHUVEC and sHUVEC. Moreover, in the sHUVEC, the expression level of miR-21 was reduced by the ZOE treatment. These results suggest that ZOE treatment can play an anti-inflammatory and a potential protective role on the vasculature.

To investigate the main constituents of ZOE involved in such anti-inflammatory and senomorphic activity, we also tested the effects of *Zingiber officinale* terpenoid-enriched extract (ZTE), as, in BV2 cells, the anti-neuroinflammatory effect appears distinctly related to this fraction. Terpenes represent one of the largest groups of plant-derived secondary metabolites, but their biological activity is poorly studied. Consistent with this, ginger oil has been scarcely investigated and only one study has reported its efficacy in reducing acute inflammation in mice [37]. Zingiberene is the most abundant component of ZTE, and it is a monocyclic sesquiterpene, mainly present in ginger with oil content (*Zingiber officinale*). ZTE has several pharmacological properties and, in particular, possesses the ability to modulate the inflammatory PI3K/AKT/mTOR pathway in human cells [38,39]. 

In our study, we demonstrated that ZTE treatment was able to significantly reduce the expression level of IL-1β and IL-8, the IL-8 secreted in the medium, and the expression of miR-21 and miR-126 in both the LPS-stimulated yHUVEC and sHUVEC, thus supporting both the anti-inflammatory and senomorphic activities of this extract. Although the anti-inflammatory activity of zingiberene has already been observed [40], the lack of data about purified zingiberene senomorphic activity is a limitation of this work and further experiments will be performed to assess the role of zingiberene in cellular senescence. 

Overall, our data show the anti-inflammatory and senomorphic effects of ZOE and ZTE on young and senescent human endothelial cells, thus supporting the efforts devoted to identifying new natural compounds with the potential ability to contribute to delaying/postponing ARD development and progression. 

## Data Availability

Not applicable.
